# Cell periphery-related proteins as major genomic targets behind the adaptive evolution of an industrial *Saccharomyces cerevisiae* strain to combined heat and hydrolysate stress

**DOI:** 10.1186/s12864-015-1737-4

**Published:** 2015-07-09

**Authors:** Valeria Wallace-Salinas, Daniel P. Brink, Dag Ahrén, Marie F. Gorwa-Grauslund

**Affiliations:** Applied Microbiology, Department of Chemistry, Lund University, P.O. Box 124, Lund, SE-22100 Sweden; Microbial Ecology Group, Department of Biology, Lund University, Ecology Building, Lund, Sweden

**Keywords:** *Saccharomyces cerevisiae*, Ethanol Red, Comparative genomics, Evolution, Regulation, Cell Wall Integrity, Lipids, Thermotolerance, Hydrolysate, Multicellularity

## Abstract

**Background:**

Laboratory evolution is an important tool for developing robust yeast strains for bioethanol production since the biological basis behind combined tolerance requires complex alterations whose proper regulation is difficult to achieve by rational metabolic engineering. Previously, we reported on the evolved industrial *Saccharomyces cerevisiae* strain ISO12 that had acquired improved tolerance to grow and ferment in the presence of lignocellulose-derived inhibitors at high temperature (39 °C). In the current study, we used comparative genomics to uncover the extent of the genomic alterations that occurred during the evolution process and investigated possible associations between the mutations and the phenotypic traits in ISO12.

**Results:**

Through whole-genome sequencing and variant calling we identified a high number of strain-unique SNPs and INDELs in both ISO12 and the parental strain Ethanol Red. The variants were predicted to have 760 non-synonymous effects in both strains combined and were significantly enriched in Gene Ontology terms related to cell periphery, membranes and cell wall. Eleven genes, including *MTL1, FLO9*/*FLO11,* and *CYC3* were found to be under positive selection in ISO12. Additionally, the *FLO* genes exhibited changes in copy number, and the alterations to this gene family were correlated with experimental results of multicellularity and invasive growth in the adapted strain. An independent lipidomic analysis revealed further differences between the strains in the content of nine lipid species. Finally, ISO12 displayed improved viability in undiluted spruce hydrolysate that was unrelated to reduction of inhibitors and changes in cell wall integrity, as shown by HPLC and lyticase assays.

**Conclusions:**

Together, the results of the sequence comparison and the physiological characterisations indicate that cell-periphery proteins (e.g. extracellular sensors such as *MTL1*) and peripheral lipids/membranes are important evolutionary targets in the process of adaptation to the combined stresses. The capacity of ISO12 to develop complex colony formation also revealed multicellularity as a possible evolutionary strategy to improve competitiveness and tolerance to environmental stresses (also reflected by the *FLO* genes). Although a panel of altered genes with high relevance to the novel phenotype was detected, this study also demonstrates that the observed long-term molecular effects of thermal and inhibitor stress have polygenetic basis.

**Electronic supplementary material:**

The online version of this article (doi:10.1186/s12864-015-1737-4) contains supplementary material, which is available to authorized users.

## Background

For many years, the improvement of microbial strains by non-targeted approaches, such as random mutagenesis, crossbreeding or evolutionary engineering, has been successfully used in situations where the knowledge of the biochemistry and genetics associated to the relevant pathways was limited, as well as when complex connections of multiple metabolic and regulatory pathways restricted the manipulation of the strains by targeted approaches [1]. However, once a superior phenotype has been obtained by a non-targeted approach, the identification of molecular mechanisms behind the improved traits becomes a challenging process. Such elucidation, however, is of great importance since it enables the re-introduction of only the relevant mutations to other strains, thereby avoiding the accumulation of negative mutations or trade-off events that may have occurred during the selection process [2]. In addition, the identification of genetic features and mechanisms behind a phenotype of interest is expected to improve our knowledge and understanding of complex biological systems.

Baker’s yeast *Saccharomyces cerevisiae* is an important protagonist in industrial biotechnology and it is considered the biocatalyst of choice for the production of ethanol from lignocellulosic biomass [3, 4]. However, the tolerance of *S. cerevisiae* to stressors encountered during the ethanol production process, such as heat, lignocellulose-derived inhibitors, salts, contaminants, among others, needs to be further improved.

Many aspects of the response of *S. cerevisiae* towards environmental stressors have already been elucidated in this model eukaryote [5–8]. Furthermore, different mechanisms by which *this yeast* responds to the different types of inhibitors that are found in lignocellulosic hydrolysate, such as furaldehydes, weak organic acids and phenolic compounds, have been also identified [9, 10]. In fact, the various reports of long-term adaptation experiments oriented to improve the tolerance of *S. cerevisiae* to a single or to a mix of hydrolysate-derived inhibitors [11–14] have helped to reveal some of the evolutionary mechanisms responsible for the superior traits in the evolved strains, notably including the up-regulation of reductases capable of the conversion of furaldehydes to less inhibitory compounds [12, 15]. In contrast, the effect of high temperature on yeast physiology has mainly been studied through heat shock experiments, that is, when cells are shortly exposed to high temperatures [16–18]. Studies on long-term adaptation of *S. cerevisiae* to high temperatures are scarce [19], and to our knowledge there are no reports on how *S. cerevisiae* evolves to survive long-term exposure to a combination of heat and inhibitors.

In the present work we further characterized an evolved industrial strain, namely ISO12, which can grow and ferment hexose sugars under a combination of stress factors (high temperature and lignocellulose-derived inhibitors) that led to complete loss of viability for the parental industrial strain Ethanol Red (ER) [20]. With the aim of uncovering possible cellular mechanisms behind the superior phenotype of ISO12, the genomic sequences of ER and ISO12 were determined and compared. In addition to this, the cell wall phenotype and lipid composition of ISO12 and ER were analysed.

## Methods

### Strains and reference sequences

The industrial *Saccharomyces cerevisiae* strain Ethanol Red [Fermentis, S.I. Lesaffre] (ER) and the derived evolved strain ISO12 [20] were used for the experiments. Cells from -80 °C glycerol stocks were maintained in YPD plates (10 g.L^−1^ yeast extract, 20 g. L^−1^ peptone, 20 g. L^−1^ glucose and 20 g. L^−1^ agar). The genome of the *S. cerevisiae* S288c strain [21] was used as a reference sequence during the analysis of the ER and ISO12 Next Generation Sequencing data. The R64-1-1 release of the S288c genome was downloaded from the *Saccharomyces* Genome Database (SGD; www.yeastgenome.org) [22] and an alternatively formatted version of this release, sacCer3, was downloaded from the UCSC Genome Browser (www.genome.ucsc.edu) [23].

### Genome sequencing, variant calling and variant analysis

#### DNA isolation

A single colony of Ethanol Red or ISO12 was used to inoculate 10 mL of YPD in a 50 mL conical tube. The overnight culture was centrifuged (2500 RCF) for 5 min, washed with 1 mL deionized water and pelleted. The cell pellet was resuspended in 0.6 mL lysis buffer (0.1M Tris pH 8, 1 mM EDTA, 100 mM NaCl and 1 % SDS final concentration). The suspension was transferred to a ready-to-use 2 mL tube containing 500 μm beads and 0.6 mL phenol/chloroform (1:1) was added. A Precellys 24 (Bertin Technologies, France) connected to a temperature controller working with dry-ice (Cryolus, Bertin Technologies, France) was used for lysis (3 cycles of 30s and 30s pause). The resulting cell suspension was centrifuged (13,000 RCF) for 5 min., and the aqueous phase was transferred to a 1.5 mL microcentrifuge tube. 200 μL chloroform was added to the tube and was mixed and centrifuged (13,000 RCF) for 1 min. The upper layer was transferred to a new microcentrifuge tube and 1 volume of isopropanol was used to precipitate DNA by centrifugation (16,000 RCF) for 20 min. The isopropanol was decanted and the pellet was washed with 1 mL 70 % ethanol, followed by centrifugation (16,000 RCF) for 5 min. The ethanol was decanted and the pellet was allowed to dry for 30-60 min. The DNA pellet was resuspended in 100 μL TE/RNAase, incubated at 37 °C for 30 min and stored at -20 °C.

#### Sequencing, assembly and variant calling

The sequencing of ER and ISO12 was performed by Genewiz Inc. (South Plainfield, NJ, USA) using Illumina MiSeq 2x250bp (Illumina Inc., San Diego, CA, USA). The *de novo* assembly of ER and ISO12 and the reference mapping and variant calling of both strains relative to S288c were performed by Genewiz Inc. using the CLC Genomics Server (v6.5.1; CLC Bio, http://www.clcbio.com). The assemblies and raw reads were deposited online, see *Availability of supporting data*. The variant calling was performed with the following thresholds: Minimum coverage (read mapping) = 10×; Minimum count of a variant = 4; Minimum frequency (count/coverage) = 25.0 %. Sequence variants that did not satisfy these criteria were discarded.

#### Analysis of the detected variants

In order to identify the variants that were unique to either ER or ISO12, all variants that were common to both strains were disregarded according to the reference mapping results. This filtering was provided by Genewiz Inc. To further predict the protein-level effects of the unique variants, the positions of the coding region SNPs and INDELs in both ER and ISO12 were reformatted from their mapped position in S288c ORFs to their corresponding chromosomal coordinates with a custom Perl (v5.10.1) script. The variants were then analysed for their non-synonymous effect on S288c ORFs using the Variant Annotation Integrator tool at the UCSC genome browser [23]. A list of the affected ORF of every detected non-synonymous variant in ISO12 was compiled and was analysed for enrichment in Gene Ontology (GO) terms by the YeastMine tool at SGD (accessed 2015-04-23). Complementary GO analyses were performed with the AmiGO 2 database [24].

The non-synonymous to synonymous substitution rate (K_a_/K_s_) [25] was calculated between the ER and ISO12 strains. Every SNP was extracted from the variant calling dataset by a custom Perl script, and was then applied to the sequences of the corresponding S288c ORFs with VCFtools (v0.1.12) [26]. The K_a_/K_s_ ratio was calculated on the modelled ER and ISO12 ORFs using the MA model of the KaKs_calculator (v1.2) [25]. The results were quality filtered to only regard values in the range of 0.01 < K_a_/K_s_ <5. It should be stressed that the K_a_/K_s_ model only accounts for polymorphisms and cannot accurately assess sequences that have changed in length during the evolutionary timeframe; therefore the INDELs were not considered for this analysis.

Non-reference material in the *de novo* assembled contigs were detected with the ABACAS software (v1.3.1) [27] coupled to the *nucmer* portion of MUMmer (v3.22) [28]. The unmapped contigs from both strains were aligned with each other with Blat (v34) [29] in order to detect non-reference sequences that were present in both assemblies. The contigs were blasted to the NCBI database (http://blast.ncbi.nlm.nih.gov) to find regions with similarities to non-reference *S. cerevisiae* strains*.*

Copy number variation was assessed for both the reference material and non-reference material. The ER and ISO12 reads were aligned to the reference genome and to the unmapped contigs with BWA (v0.7.12) [30], were compressed and sorted with SAMtools (v1.1) [31] and were finally analyzed for variation in copy number with CNV-seq (2014/08/12-version) [32] and R (v3.1.0) [33]. For CNV-seq, the genome size parameter was set according to the size of the reference data.

### Lipidomics

#### Cell cultivations for lipid analysis

Cells samples for analysis of lipids were obtained from aerobic continuous cultures. Inoculum was prepared by growing the cells overnight in 500 mL shake flasks containing 50 mL defined mineral medium [34] buffered with phthalate buffer (pH 5.5). After centrifugation and a washing step with deionized water, the pellet was resuspended with 20 mL of defined mineral medium and used for inoculation of the bioreactor. Chemostat cultivations were carried out at 30 °C in 0.75 L Multifors bioreactors (Infors, Switzerland) with a working volume of 450 mL. Aerobic conditions were maintained with an air flow rate of 1 vvm and stirring at 600 rpm. The pH was controlled at 5.5 by addition of 3M KOH. Defined mineral medium with 10 g. L^−1^ glucose was fed at the end of the batch phase with a dilution rate of 0.1 h^−1^. Carbon dioxide in the exhaust gas was measured with a 1313 Fermentation monitor (Innova, AirTech Instruments, Denmark). Steady state conditions were obtained after five volume changes (confirmed by CO_2_ analysis in the exhaust gas). Samples for biomass determination and High Performance Liquid Chromatography (HPLC) analysis were obtained from 6 independent steady states, four of which were independent biological replicates.

Quenching of the samples was achieved by quickly adding 20 mL of cell culture to 30 mL cold methanol (83.3 % methanol in water) in pre-cooled 50 ml conical tubes. After immediate mixing, the cells were cooled in a dry ice-ethanol bath for maximum 20 s and harvested in a pre-cooled centrifuge (-9 °C; 1500 RCF) for 5 min. The supernatant was discarded and residual medium was removed with a pipette while working over dry ice-ethanol bath. A second washing of the pellet was done with 10 mL cold-buffered methanol (60 % in water). The cells were collected as before and the samples free of residual medium were frozen in liquid nitrogen and stored at -80 °C.

Cell dry weight determinations were performed at each steady state by triplicate measurements. For this, 5 mL of the culture were filtered through pre-weighted nitrocellulose filters (0.45 μm; Pall Corporation, NY, USA) washed with distilled water and dried for 8 min in a microwave (350 W). Quantification of extracellular substrates and products was done with Waters HPLC system (Mildford, MA, USA). An Aminex HPX-87H ion exchange column (Bio-Rad, Hercules, CA, USA) was used for separation and a refractive index detector (RID-6a, Shimadzu, Kyoto, Japan) was used for detection. 5mM H2SO4 was used as mobile phase at a flow rate of 0.6 mL.min^−1^ and the column temperature was 45 °C.

Samples of the continuous cultures were also used to inoculate 500 mL shake flasks containing 50 mL of spruce hydrolysate (whose composition is described below) diluted to 50 % (v/v) with mineral medium [34], and incubated at 39 °C. Evaluation of growth under these conditions was carried out by optical density measurements at 620 nm (OD620) as previously described [20].

#### Extraction and quantification of lipids

Extraction and lipid quantification were carried out at Metabolomic Discoveries GmbH (Potsdam, Germany). For lipid extraction 40 mg of quenched cells were mixed with chilled 1:2.5:1 water:methanol:chloroform. Additionally, cells were mechanically disrupted in a ball mill. Cellular debris was removed by centrifugation. 1 mL of supernatant was mixed with 750 μL water. After phase separation, the lower non-polar phase was evaporated and resuspended in methanol. The LC separation was performed using hydrophilic interaction chromatography with Accucore HILIC (Thermo Fisher Scientific Inc., Waltham, MA, USA), operated by an Agilent 1290 UPLC system (Agilent, Santa Clara, USA). The LC mobile phase was A) acetonitrile and B) 0.1 % (v/v) formic acid in water with the following gradient: 1 to 6 min increase of B) from 3 % to 15 %, 6 to 7 min increase to 50 % and flush for 1 min, subsequently equilibrate. The flow rate was 500 μL.min^−1^, injection volume 1 μL. The mass spectrometry was performed using a 6540 QTOF/MS Detector (Agilent, Santa Clara, USA) with electrospray ionization. The measured metabolite concentration was normalized to internal standards.

Due to the small lipid phase obtained in two of the biomass samples of ER, the analysis of the lipid content for this strain was done only with four samples. In the case of ISO12, one of the samples was identified as an outlier and excluded from the study; therefore the analysis of the lipid content of this strain was done with five samples. Significant changes of metabolites were analysed considering variance homogeneity and p-values below 0.05 were considered as significant. The difference in the concentration of the lipid species is reported as log2-ratio of ER/ISO12.

### Physiological characterisation

#### Growth on non-fermentable carbohydrates

Mitochondrial function was evaluated by growth on glycerol and ethanol as sole carbon sources [35]. For this, YPD-grown cells of ER and ISO12 were used to inoculate YNB medium supplemented with 3 % (v/v) ethanol as carbon source or were diluted with deionized water and plated on YPG plates (10 g.L^−1^ yeast extract, 20 g.L^−1^ peptone, 3 % (v/v) glycerol, 20 g.L^−1^ agar) and incubated at 30 °C for 48h. YPDG plates (10 g.L^−1^ yeast extract, 20 g.L^−1^ peptone, 1 g.L^−1^ glucose, 3 % (v/v) glycerol, 20 g.L^−1^ agar) were used to determine the proportion of petite mutants that lacked or had defective mitochondrial DNA (*rho*^*−*^ cells). Growth on YPD plates was set as control conditions.

## Results and discussion

### Characterisation of the genome-level effect of the ISO12-adaptation

#### Genome sequencing, assembly and variant calling

The genomes of the Ethanol Red (ER) and ISO12 yeast strains were sequenced using the Illumina Next Generation Sequencing (NGS) platform. This resulted in 15M and 12M reads for ER and ISO12 (NCBI Sequence Read Archive: SRR2002842 and SRR2002960), both displaying a median PHRED score of 38 (corresponding to a 0.02 % base call error rate [36]). The reads were mapped to the *S. cerevisiae* S288c reference genome (average coverage: ER 185×; ISO12 153×) and the sequence variants relative to the reference strain were detected for both strains. Furthermore, the reads were also used to *de novo* assemble the genomic sequences of ER and ISO12 in order to assess the effect of the adaptation in genomic material not found in the reference strain. The ER assembly resulted in 218 contigs (average coverage: 301×) consisting of a total of 11.5 Mbp (GenBank: JWJK00000000). For ISO12, 361 contigs were assembled (average coverage: 196×) with a total size of 11.4 Mbp (GenBank: LBNP00000000). A summary of the sequencing, reference mapping and *de novo* assembly results is found in Table 1.

**Table 1:** Sequencing, reference mapping and *de novo* assembly results (as reported by Genewiz Inc.)

Sequencing	Count	
	Ethanol Red	ISO12
Total no. of reads	15,458,851	12,449,273
Median PHRED score	38.0	38.0
Mean GC content	38 %	38 %
**Reference mapping to the S288c reference sequence**	Count	
	Ethanol Red	ISO12
No. of mapped reads	15,341,613 (99.2 %)	12,353,009 (99.2 %)
Average coverage	185×	153×
***De novo*** **assembly**	Count	
	Ethanol Red	ISO12
No. of matched reads	15,390,426 (99.6 %)	12,195,728 (98.0 %)
Average read length (bp)	147.6	151.1
Total contigs assembled	218	361
Total bases in contigs	11,492,342	11,356,874
Average coverage	301×	196×
N50 (bp)	95,106	67,943

The variant calling was set to identify two types of variations - SNPs (Single Nucleotide Polymorphisms) and INDELs (Insertions and Deletions) - and resulted in 78,925 (ER) and 78,143 (ISO12) variants relative to S288c (Table 2), using the minimum thresholds of detection stated in the Materials and Methods section. This was an expected magnitude, similar to a previous study that also mapped a diploid strain to S288c [37], and it reflected the marked differences between the genomes of different strains in general, and between the genomes of laboratory and industrial strains in particular [38, 39]. Since the adapted strain was derived from ER by laboratory adaptive evolution [20], a high number of the identified variants versus S288c (76,661) were found to be common to both strains. These SNPs and INDELs were disregarded, and further analyses focused on the remaining 2,264 variants that were found only in ER and 1,482 variants that were unique to ISO12.

**Table 2:** Results of the variant calling and -analysis

Variants (SNPs and INDELs)	Count	
	Ethanol Red	ISO12
General statistics		
Number of variants detected versus S288c	78,901	78,133
Number of variants unique to strain	2240/78,901	1472/78,133
Statistics of the strain-unique variants		
Intergenic variants	1293/2240	969/1472
Coding region variants	947/2240	503/1472
Predicted effects of the coding region variants^a^		
Synonymous effects	536	222
Non-synonymous effects	454	306

^a^Including overlapping ORFs

Since only around 300 generations separated ISO12 from its parental strain, the number of identified variants suggested a higher mutation rate per genome than previously reported [40]. One possible reason may be an underestimation of the number of generations for ISO12, since the decrease in the number of viable cells due to the cytotoxic effects of hydrolysate and temperature was not taken into consideration every time a new batch was inoculated [20]. Also, the number of alterations reported in other evolutionary experiments using *S. cerevisiae* shows significant differences. For example, the number of mutations reported for *S. cerevisiae* CEN.PK113-7D after over 300 generations growing at high temperature (40 **°**C) was of only 30 SNPs distributed in 18 genes [19], while a total of 949 alterations were reported for S288c after approximately 140 generations during serial transfers with increasing concentrations of barley hydrolysate [41]. These results suggest that the mutation rate will be affected by the genomic stability of the background strain as well as by the type of stress encountered during the evolutionary experiment. Therefore, apart from a probable higher natural mutation rate in ER compared to the aforementioned strains, it is very likely that the stressful environment - given by the combination of high temperature and toxic hydrolysate-derived compounds- significantly increased the rates of random mutagenesis; an effect referred as “stress-inducible mutability” that has been discussed in previous studies [42, 43]. Also, the diploid nature of these strains possibly allows for a greater accumulation of mutants than a haploid strain would [42, 44] because undesired mutations would not be selected against and could thus be inherited and add to the accumulation of mutations as long as one functional wild type allele remains in the genome. Finally, an inevitable possibility when working with NGS data is that of erroneous data interpretation due to sequencing error. For Illumina data, a number of technical difficulties on the sequencing-level have been proposed to increase the false SNP call rate, including under-representation of GC-rich regions during the PCR-amplification step [45] and strand bias (differing genotype on the forward and the reverse strand) during read mapping [46]. In the end, we cannot rule out that phenomena like these might have affected the false positive count in the dataset. It should be mentioned that we, in addition to the reference-mapping approach, also attempted variant calling by mapping ISO12 reads to the ER *de novo* assembly. However, this resulted in an even higher number of variants between the two strains(~9000; data not shown), which might be due to a high rate of false positive variant calls. We thus believe that the method that is presented here (reference-mapping) was a sound choice for this study.

### Analysis of the distribution and the protein-level effects of the sequence variants

The SNPs and INDELs detected in ER and ISO12 were found to be distributed throughout the extent of the S288c reference genome, albeit with varying peak density (Additional file 1: Figure S1). In fact, circa 60 % of the detected variants in both ER and ISO12 were intergenic (Table 2). Since only ~27 % of the *S. cerevisiae* genome is non-coding [47], this result indicated a heavy bias towards intergenic alterations during the evolution of ER into ISO12. Many investigations have shown that variations in non-coding regions have an important impact on phenotypic diversity given their influence on gene expression level [48]. Although the differences in intergenic regions would definitely merit further investigation, for the purpose of the current study we focused the analysis on variants within coding regions.

The Variant Annotation Integrator web-tool [23] was used to identify and classify the protein-level effects of the SNPs and INDELs. The non-synonymous mutations affecting coding regions (including overlapping ORFs) were distributed in the following manner: 454 non-synonymous SNPs were allocated in 221 ORFs in ER (Table 2; Additional file 2) while 306 non-synonymous SNPs were distributed among 172 ORFs in ISO12 (Table 2; Additional file 3). Forty-eight ORFs presented variants in both ER and ISO12, while 173 and 124 ORFs presented SNPs only in ER and ISO12 respectively. Altogether, ER and ISO12 presented genetic variations in a total of 347 different ORFs (Additional files 2 and 3). A majority of the non-synonymous variants were classified as missense variants, i.e. changing a single amino acid but not affecting protein length or read frame (88 % in ER; 78 % in ISO12). Furthermore there were 83 variants in both strains that were predicted to generate a premature stop codon or a frame shift, and could therefore result in loss of protein function (7 % in ER; 16 % in ISO12).

To investigate the 347 genes that were predicted to have gained non-synonymous alterations, we applied a number of complementary analyses. A smaller number of candidate genes with high relevance to the novel phenotype of ISO12 was identified by assessing functional annotation enrichment and haplotype variation (homo-/heterozygosity; cf. annotations in Additional files 2 and 3), as well as by modelling of the non-synonymous to synonymous substitution ratio (K_a_/K_s_) and copy number variation (CNV). These approaches are elaborated in the following sections.

#### Enrichment in cell periphery-related proteins

**Fig. 1:**
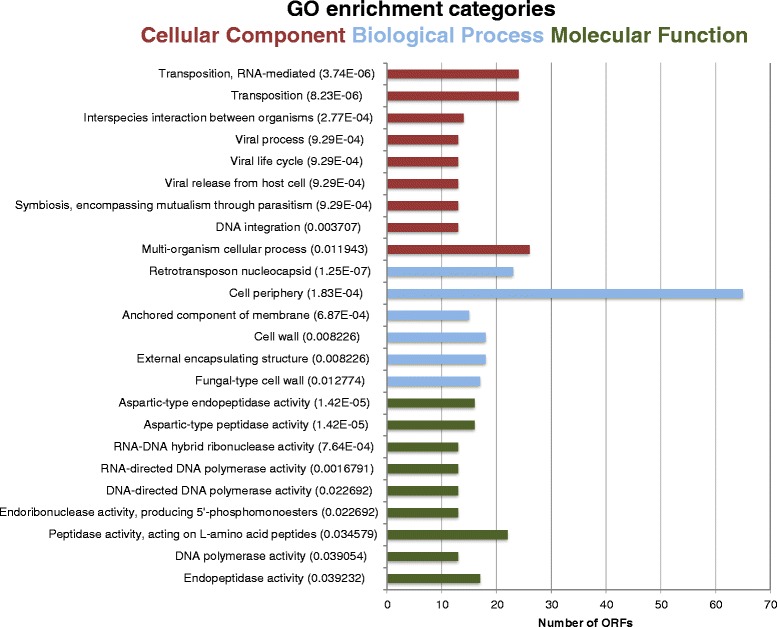
Significantly enriched Gene Ontology (GO) terms for the non-synonymous variants detected in ISO12 compared to ER (p < 0.05, Holm-Bonferroni corrected). Terms were sorted in three categories: *Biological process*, *Cellular component* and *Molecular function*. The enrichment analysis was performed using the YeastMine at *Saccharomyces* Genome Database (SGD). Note that the same ORF can figure in multiple bars; please refer to Additional file 4 for the full list of ORFs

The 347 ORFs that differed between the strains were first investigated-independently from ploidy, substitution ratio and CNV-for their enrichment in Gene Ontology (GO) terms using SGD (p < 0.05, Holm-Bonferroni corrected; background: SGD default) in order to infer cellular functions and structures that were overrepresented (Fig. 1). By default, the tool at SGD assesses GO enrichment in three different categories: *Biological process*, *Cellular component* and *Molecular function*.

In the *Biological process* ontology, transposition, RNA mediated (p = 3.74x10^−6^; 24 matching ORFs) and interspecies interaction between organisms (8.24x10^−6^; 14 matching ORFs) were the most significantly enriched terms (Fig. 1). 36 different ORFs were represented in the 9 enriched terms obtained within the category *Biological process* (Additional file 4). Of these 36, 24 were directed related with transposition-associated processes (transposable-element-gene) while the remaining 12 ORFs were associated with cellular processes such as flocculation (markedly the *FLO* gene family), agglutination, conjugation and fusion (Additional file 4).

**Fig. 2:**
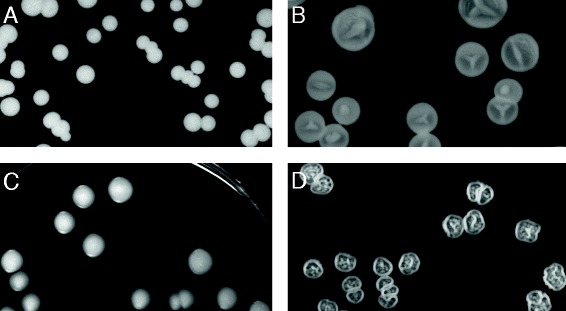
Colony morphology of ER (**a** and **c**)) and ISO12 (**b** and **d**) when grown on glucose (**a** and **b**) and glycerol **c** and **d**. For growth on glucose the picture shows the bottom of the colony, while the surface is presented for growth on glycerol

With respect to the *Cellular component* category, the enrichments revealed that around 20 % of the ORFs differing between ER and ISO12 encoded proteins related with structural components of the cell such as the cell wall and the plasma membrane and with the assembly and organization of the cytoskeleton (Fig. 1). The two most enriched terms in this category corresponded to the retrotransposon nucleocapsid (p = 1.25x10^−7^; 23 matching ORFs) and cell periphery (p = 1.83x10^−4^; 65 matching ORFs); in fact, the cell periphery category was the GO term that was enriched in the highest number of ORFs (Fig. 1). Some of the molecular functions covered by the cell wall-associated ORFs included heat shock protein (*HSP150*), transcription factor (*RPI1*) and components of the cell wall integrity signalling pathway (*SPA2)*. Sequence variations were also found in ORFs coding for various glycosylphosphatidylinositol (GPI)-anchored cell wall proteins (e.g. *EGT2*, *YPS1*, *YPS6*, *FIT1*). Moreover, ER and ISO12 differed in the genomic sequence of several ORFs coding for proteins involved in cellular surface properties such as adhesion (*AGA1* and its paralogous gene *FIG2*), and biofilm and flocculation (*FLO1*, *FLO5*, *FLO9*, *FLO11*) (Additional file 4). SNPs related to membrane proteins included ORFs coding for various type of transporters such as the ABC-transporter *PDR5*, the multidrug efflux transporter *ATR1*, the iron transporter *FTR1* and the proton-gluthatione transporter *GEX1*, among others. Notably, 5 of the at least 20 hexose transporters present in *S. cerevisiae* [49] presented genomic differences between the strains (*HXT3*, *HXT4*, *HXT6*, *HXT13* and *HXT15*). Also, gene variations were found in ORFs whose transcription products are associated with the sensing and reception of nutritional signals (*GPR1*, *KOG1*) and oxidative stress (*MTL1*). Finally, the genomic variations identified in sequences coding for proteins involved in the assembly and organization of the cytoskeleton included SNPs in *ABP1*, *SLA1*, *NUM1*, *BEM3* (Additional file 4).

It is well established that cell wall and membranes are highly dynamic structures that respond to various forms of environmental stress [50–52]. Heat in particular has been shown to reduce membrane potential and disrupt the membrane integrity, affecting its permeability and fluidity [53, 54]. The enrichments of genes directly related with cell wall and membrane indicate therefore that such structures were important targets during the evolutionary experiment. Although some genomic variations may be associated with the deleterious effect of the stressors over these cellular components, the direct relationship of some of the aforementioned proteins (or similar ones) with the stress tolerance response in yeasts [55, 56], suggest that variations in the regulation, structure and/or activity of these protein variants as a result of the point mutations and other genomic alterations, were necessary for the cell to adapt to the severe conditions. In fact, ISO12 exhibited an improved viability during 48h-cultivation in undiluted spruce hydrolysate while only little or no reduction of inhibitors was observed (Additional file 1). We hypothesise that this results from changes in membrane permeability that were induced by long-term exposure to stressors. Similarly, the already reported negative effects of high temperature and furfural over cytoskeleton elements and consequently over their physiological functions - including vesicular transport- may explain the alterations in more than 6 ORFs related with the cellular matrix [57–59]. Actually, according to the AmiGO 2 database [24], 20 ORFs associated with vesicle-mediated transport presented genetic variations between the strains, e.g. *SEC7, SPO14* and *SED4* (Additional file 5). However, bearing in mind that ISO12 is indeed capable of growing and fermenting under the combination of the stresses, the results suggest that either such alterations were phenotypically neutral or that at least some of the new variants of the actin-related proteins in ISO12 would contribute to the adaptation response, among other, by allowing the cell to carry on with crucial intracellular traffic of proteins.

The enrichment of genes in the third GO category, *Molecular function* covers different types of proteases. Of particular relevance are the ORFs coding for proteasome sub-units and ubiquitin-specific proteases that are present in both the nucleus and cytoplasm (*RPN2*, *UBP8*, *UBP10*, *UBP12*) (Additional file 4). Recently it was demonstrated that a single mutation in the ubiquitin-specific protease gene *UPB7* was directly correlated with tolerance to spent sulphite liquor, a raw material for ethanol production with similar type of inhibitors as spruce hydrolysate [60]. Similarly, other studies have established the effect of mutations in different proteases on the response of the cell to different environmental effectors [61, 62]. The SNPs identified in ISO12 emphasize hence the relevance of the ubiquitin-mediated protein homeostasis machinery during heat and inhibitor stresses. The most enrichment terms in this ontology corresponded to aspartic-type endopeptidase activity (p = 1.42x10^−5^; 16 matching ORFs) and RNA-DNA hybrid ribonuclease activity (p = 7.64x10^−4^; 13 matching ORFs; Fig. 1).

The enrichment of transposition-related ORFs in all the three categories of the gene ontology database suggest that changes in the DNA sequences of retrotransposons are a constant feature behind the genetic differences between ER and ISO12. On the one hand, retrotransposons (Ty elements) have been clearly identified as responsible entities for evolutionary genomic and phenotypic changes in *S. cerevisiae* strains by causing gene disruption, promoting chromosomal rearrangements or acting as regulatory agents [63, 64]. On the other hand, the results obtained in the current study do not provide information about the transpositional activity of the Ty elements, and therefore it is not possible to establish a direct relationship between the high numbers of GO enrichments for Ty elements related events with phenotypic differences between the strains. Yet, considering the diversity of variants in the Ty coding regions (Additional files 2 and 3), we speculate that at least some of such SNPs may be responsible for a higher rate of replication of the transposon and/or for providing an adaptive advantage to ISO12 under the stress conditions.

#### Eleven genes were found to be under positive selection in ISO12

The GO analysis revealed that the dataset with the ORFs with SNPs/INDELs were enriched in terms related to cell-periphery proteins. Next, we investigated each gene in this dataset by looking at which ORFs that were considered likely to be evolved under positive selection, i.e. a directional selection driven by change in phenotype [65]. The non-synonymous to synonymous substitution ratio (K_a_/K_s_) [25] was calculated between the two strains based on the effect the SNPs would have on a S288c background (Additional file 6). 11 genes were found to be under positive selection (K_a_/K_s_ >1). Although GO enrichment analysis is not applicable on this small dataset, a relationship similar to that of the above GO enrichment analysis was found. The proteins linked with these eleven ORFs were found in different locations within the cell; *MTL1* (K_a_/K_s_ = 3.2), *HXT13* (K_a_/K_s_ = 4.1), *CYC3* (K_a_/K_s_ = 3.1) and *SED4* (K_a_/K_s_ = 1.5) code for proteins associated with membranes; *FLO9* (K_a_/K_s_ = 2.3) and *FLO11* (K_a_/K_s_ = 1.3) are linked to the cell wall and *SRP40* (K_a_/K_s_ = 1.2) with the nucleus. The four other ORFs are associated with proteins of unknown/uncharacterized location and function. It can be noted that *SRP40* and the four putative ORFs did not show up during the GO enrichment analysis while the other ORFs did (Additional files 4 and 5). However, it should be kept in mind that the enrichment assessment only takes functional annotations and not the number of mutations into consideration, and in that sense K_a_/K_s_-modelling complements the enrichment analysis.

The implications of the stress-membrane sensor Mtl1p [66] as part of the cellular response to oxidative stress are physiologically coherent with the conditions of the evolutionary experiment [20]. *MTL1* is a partly redundant paralog of the major cell wall integrity (CWI) sensor *MID2* and is involved in the oxidative stress response and actin cytoskeleton repolarisation [66] and has also been named a putative sensor for glucose starvation [67]. Furfural - one of the stressors that were present during the ISO12 adaptation - has been reported to cause accumulation of reactive oxygen species (ROS) that, among others, damages the actin cytoskeleton [57]. In *S. cerevisiae* the CWI pathway functions by a Mitogen-Activated Protein Kinase (MAPK) cascade that leads to the activation of transcription factors controlling the expression of genes responsible for adjusting cell wall structure and composition according to the environmental input, and *MTL1* is one of the sensors that can initiate the cascade in response to cell wall stress [50, 68]. The apparent positive selection of *MTL1* is thus highly interesting, and since the identified variants in this ORF were solely missense variants and not loss-of-function variants (such as stop and frameshift variants) we hypothesize that the CWI sensing in the adapted strain might have undergone functional alterations. However, when the CWI of ER and ISO12 were evaluated experimentally with an enzymatic cell wall lysis assay (lyticase) no significant differences in maximal lysis rate were observed (Additional file 1). From this it was concluded that there had been no changes in β 1,3-glucan integrity, which however does not rule out other changes to the CWI system. In fact, it has been reported that β-1,3-glucanase (here: lyticase) is sensed with combined signalling of the high osmolarity/glycerol pathway and the CWI, but that upstream CWI elements such as *MTL1* does not contribute to the response [69]. This could explain why the detected alterations in *MTL1* were not reflected in the results of the lyticase assay.

The *FLO* (flocculation) genes also stand out as important targets during the evolution. First, mutations in the *FLO9* and *FLO11* ORFs appeared to have evolved under positive selection (Additional file 6). Second, the sequence regions coding for the flocculation-related genes *FLO1* and *FLO11* showed homozygous SNPs in both strains (Additional files 2 and 3), and finally around 25 SNPs between the strains were found in the genome sequence of *FLO5*. This means that 4 out of the 5 *FLO* genes present in *S. cerevisiae* [70] differed between strains*.* It is known that changes in sequence, copy number transcriptional level and other regulatory mechanisms of the *FLO* genes affect several of the adaptive mechanisms of the cells towards detrimental environmental factors including flocculation, biofilm formation and agar invasion [71–73]. In fact, previous studies have shown direct evidence of the role of *FLO* genes on the adaptation capacity of yeast cells to overcome temperature or lignocellulosic-derived inhibitors [74, 75]. The genotypic variations between ISO12 and ER in the *FLO* genes correlated with a higher capacity in ISO12 to form biofilms (data not shown), invade agar and form novel colony morphologies under certain conditions (See *Growth on non-fermentable substrates and multicellularity* section below). It seems therefore very reasonable that the acquired properties of ISO12 cells to aggregate, mediated by at least some of the *FLO* genes variations, are part of the cellular adaptive mechanisms to resist the combination of both stresses.

Other genes predicted to have evolved under positive selective pressure were *CYC3* and *HXT13* (Additional file 6). *CYC3* is a nuclear gene that codes for cytochrome c heme lyase. *cyc3* null mutants are respiratory deficient since they lack a functional cytochrome c, and their ROS levels have been found to be lower than wild-type strains [76, 77]. Although the missense variant present in ISO12 (M45I; detected in 38 % of the mapped alleles) is most probably functionally active, the effect of such amino acid change on the decreased respiratory capacity of ISO12 (by direct effects on CYC3p or through epigenetic mechanisms) cannot be discarded [78]. In relation to *HXT13*, it has been proposed that down-regulation of hexose transporters as a mechanism to reduce growth could be part of the cellular response during heat-shock [79]. *HXT13* is not considered a main yeast hexose transporter [80] and the impact of the changes to *HXT13* is in itself not very evident, however, other hexose transporters did exhibit mutations in ISO12, including a frameshift INDEL in *HXT4* (detected in 27 % of the mapped alleles).

The significance of the other six genes that appeared to have evolved under positive selection is less clear.

#### Haplotype- and copy number variations indicate possible gene dosage effects in ISO12

An increase in the relative content of heterozygous variants was observed in the datasets of both strains after subtraction of the common variants. Approximately 16 % of the total SNPs/INDELs were classified as heterozygous in both original datasets. This changed to 76 % in the ER dataset and to 83 % for ISO12 after the shared variants were disregarded. Due to the polyploid nature of the strains, the high content of heterozygous variants in the datasets implies that the possible novel effects of many of the strain-unique variants in ISO12 might be overshadowed by parallel wild-type alleles. On the other hand, heterozygous variants can also confer haploinsufficiency in yeast [81] (particularly affecting protein complexes due to imbalances in subunit availability [82]) and thus haplotype variations remain relevant to the ISO12 case.

It was found that loss-of-function variants such as premature stop codons and frame shifts were almost exclusively heterozygous in both strains, with no homozygous calls in ISO12 and seven in ER. It is nevertheless possible that the predicted novel stops in 15 genes in the adapted strain (Additional file 3) is contributing to the phenotype by gene dosage effects. An intriguing example in the present study is the premature stop in the external glucose sensor *GPR1* (detected in 59 % of the *GPR1* alleles in ISO12 through reference mapping). Gpr1p belongs to the RAS-cAMP-PKA pathway and constitutes the means of which this pathway reacts to extracellular glucose [83]. Down-regulation of the RAS-cAMP-PKA pathway has been associated to decreased heat sensitivity [84] – a prominent phenotype in ISO12- as decreased Protein Kinase A (PKA) levels induces transcription of a number of stress response genes with *stress response elements* (STREs) in their promoter regions [85]. The premature stop codon in Gpr1p was predicted in amino acid 251 of 961 that, in addition to the deprivation of three quarters of the protein, implicates a complete loss of the cytoplasmic tail [83] which is highly likely to cause a disruption in the signal transmission. However, to fully elucidate this, cAMP/PKA levels would have to be measured and compared between the adapted and the parental strain.

Gene dosage effects can furthermore affect phenotype not only by changes in haplotype but also by copy number variation (CNV). CNVs are, in addition to SNPs and INDELs, a common genomic evolutionary effect and have been observed both in laboratory adaptation experiments [86, 87] and in natural isolates and industrial yeast strains [38, 88]. We used a reference mapping-based method [32] to assess the changes in copy number between ER and ISO12 and detected 13 regions with a >2-fold higher CNV and 77 regions with a >2-fold decrease in ISO12 (Additional file 7). As 46 % of the CNV-regions were located either in telomere or transposon regions, they are likely to be false CNV-calls as centromeric and telomeric regions are known to be highly difficult to sequence and assemble/reference-map due to their enrichment of tandem repeats [89]. When a log2 threshold was applied, 32 of the CNV-regions were found to correspond to ORFs (excluding telomere and transposon regions), including a two-fold increase in *FLO9* (p = 4.65E-31) and *ENA1* (p = 3.78E-61) (Additional file 7). Three ORFs exhibited a CNV higher than three-fold: *ENA2* had increased from ER to ISO12 (log2 = 3.0, p = 7.68E-206), and two ORFs, *PHO12* (log2 = -2.2, p = 69E-83) and *FLO1* (log2 = -3.7, p = 8.88E-126), had decreased.

Sequencing of *PHO12* has been previously been proven problematic due to the presence of paralogs [39] and for this reason we cannot rule out the possibility that *PHO12* is subject of a false CNV-call. But the CNVs in the *FLO* genes matched the above results regarding positive selection pressure and bring additional evidence to support the correlation of alterations in this gene family and the phenotype of ISO12 (cf. also the discussion regarding the effect on CNVs in *FLO* genes in the previous section on K_a_/K_s_). As for *ENA1* and *ENA2*, they encode ATPase sodium pumps [90], and results presented by Gilbert et al. showed that an increased copy number of *ENA1*, *ENA2* and *ENA5* genes in two different lines of mutants strains obtained after evolution under increased concentrations of acetate, was associated to improved acetate and temperature robustness [91]. The similarity on the CNVs in the *ENA* genes in ISO12, whose adaptation was also carried out in the presence of acetic acid and at elevated temperature, supports a relationship between the function of the *ENA* genes and these two types of stressors and suggest that such genes have a tendency to CNVs as a common stress response in yeast.

#### Assessment of the non-reference genomic material through *de novo* assemblies

Industrial yeast strains often contain genomic material that is not shared with the S288c reference strain due to differences in their lineage [38, 88]. These variations in genome content can confer important strain-specific geno- and phenotypes and assessment of such regions will add to the larger picture of evolutionary effects. Again, we focused on investigating the changes between the parental and adapted strain. By mapping the contigs from the *de novo* assemblies to the reference genome, we found 8 contigs in ER and 13 in ISO12 that could not be mapped to the reference (listed in Additional file 8; GenBank JWJK00000000 and LBNP00000000). Alignment of the unmapped contigs with each other revealed that there were five alignment pairs that were present in both strains; in total, 81.5kb were successfully aligned (Additional file 8). The top blastx-results showed (as was hypothesised) that there were regions in these contigs with high similarities to protein-coding regions from non-S288c strains, including the wine strain AWRI796, the industrial bioethanol strain-derivative JAY291 and the sake brewing yeast Kyokai No. 7.

In contrast with the CNV-analysis of the reference-material, the majority of the CNVs detected in these contigs were higher in ISO12 than in ER: in total, 33 regions with >2-fold CNV was detected in these five pairs, of which 29 were higher in ISO12 (Additional file 8). The largest contigs in terms of size (ER contigs 43 and 90) did also contain the highest density of CNV-regions. Using blastx, we could identify that a two-fold increase for ISO12 in a region corresponding to *ADH7* from the YJM1447 strain (99 % identity), and a 2-fold decrease in ISO12 in the loci with similarity to *AAD10* from strain AWRI796 (97 % identity) and *UIP3* from JAY291 (99 % identity). These genes are present also in S288c, however, the results of the blast search resulted in higher identities with the non-reference strains, which suggests that the lateral sequences were different. When blasted towards SGD, ER Contig 90 displayed high identity to mitochondrial material from non-S288c *S.cerevisiae* strains; however, none of the CNV-regions matched any annotated ORFs from the database.

The proposed increase in CNV for *ADH7* is especially intriguing in the context of the novel ISO12 phenotype. Increased *ADH7* activity has previously been positively correlated with HMF tolerance; upregulation of this gene has been observed in HMF tolerant yeast strains [92, 93] and overexpression of *ADH7* has been shown to result in a significant increase in HMF reduction capacity with NADH as cofactor and also improved cell growth recovery in HMF [94]. However, this is a surprising find in relation to the actual ISO12 phenotype as the HMF-reduction capacity in this strain was found to be lower than in ER [20].

### Lipidome comparison

Lipids play a key role on the biological functionality of membranes, by providing a permeable barrier and by maintaining its liquid crystalline state [95]. The effects of environmental stressors on cellular membranes have been extensively studied due to the essential role of these structures for proper functioning of the cell. In general, the consequences of environmental conditions on membranes are described by perturbations of the membrane fluidity, i.e. alterations on membrane composition and organization that not only affect the permeability of the barrier (e.g. leakage), but also disrupt the functionality of many membrane-associated proteins (e.g. transporters), and influence the expression of many genes (e.g. heat shock genes) [53, 96]. Lipids are of primary relevance for membrane functionality at high temperatures; in fact, it has been suggested that the reason why there are no known extreme eukaryotic thermophile can be attributed not only to their susceptibility to mRNA degradation, but also to the susceptibility of their lipids to changes in permeability and phase-state when exposed to high temperatures [97].

Alterations in lipid-membrane composition are known to take place as a response to changes in temperature and chemical stressors [98–102]. Based on the above, and in parallel to the genomic analysis, the differences in lipid composition between ER and ISO12 were analysed by a relative quantitative approach (i.e. the lipids species of one strain were compared in relation to their levels in the other strain). The lipidomes of the two strains reflected important differences in more than one lipid species (Table 3). Nine out of 119 analysed lipid species were found in relative higher concentration in ISO12 with all lipid classes represented by at least by one metabolite (p = 0.05, fold change > 2). The raw data of the lipid analysis from all the samples and the differential analysis are available in Additional file 9. Although the relevance of the particular lipids is difficult to assess, it is pertinent to note that accumulation of cerotic acid has been reported as part of a mechanism of cell resistance to oxidative stress [103]. Considering that during the evolutionary process both heat and some of the hydrolysate-derived inhibitors were a source of reactive oxygen species (ROS) [57], alterations in the content of cerotic acid may account, at least in part, for the increased resistance in ISO12. There was no significant difference in the concentration of ergosterol between both strains, but a higher concentration of ergosta-5,7,22,24(28)-tetraene-3beta-ol –the direct precursor of ergosterol– was found in ISO12. Changes in sterol composition have been found to be associated with higher thermotolerance in yeasts [19, 104, 105]. Considering that in terms of biological functionality ergosta-5,7,22,24(28)-tetraene-3beta-ol and ergosterol are very similar under normal conditions [106], the increase of ergosta-5,7,22,24(28)-tetraene-3beta-ol in ISO12 may be linked to the adaptive mechanisms developed by the cells to survive the high temperature. Similar changes in lipid composition are also likely to confer the improved cell viability in undiluted spruce hydrolysate of ISO12, that was found to be unrelated to inhibitor detoxification (Additional file 1).

**Table 3:** Significantly enriched lipids in ISO12 compared to ER

Metabolite	ER/ISO12 (Log2 ratio)	Lipid class
Hexacosanoic acid (Cerotic acid)	−1.7620	Fatty Acids
Ergosta-5,7,22,24(28)-tetraene-3beta-ol	−1.6764	Sterols
1-hexadecanoyl-sn-glycerol	−1.0575	Glycerolipids
1-octadecanoyl-rac-glycerol + 0.634	−1.8446	Glycerolipids
1-eicosanoyl-2-docosanoyl-sn-glycerol	−1.3991	Glycerolipids
GPEtnNMe(18:1(9Z)/18:1(9Z)) + 4.829	−1.3707	Glycerophospholipids
1,2-di-(9Z-octadecenoyl)-sn-glycero-3-phospho-(1'-myo-inositol)	−1.3500	Glycerophospholipids
1-octadecanoyl-2-(9Z-octadecenoyl)-sn-glycero-3-phosphoserine	−1.3625	Glycerophospholipids
CerP(d18:0/16:0)	−1.0380	Sphingolipids

The lipidomic results prompted us to look at the genomic differences associated with lipid metabolism. By matching the 414 ORFs recognized under the category *lipid metabolic process* (cf. AmiGO 2 database) with the 347 ORFs differing between the strains, 25 ORFs were identified (Additional file 5). The non-synonymous variants were identified in genes related with biosynthesis, regulation and modification of the main lipid classes fatty acid (*EHT1*), glycerophospholipid (*SLC1*, *NTE1*, *GPI12*, *FAB1*), sphingolipid (*SKN1*, *TOR1*, *LCB4*) and sterol (*HMG1*, *ERG13* and *MOT3*) but it was not possible to directly correlate the effect of the genetic variations and the pathways associated with the metabolism of the altered lipid species. Nevertheless, both types of evidence point towards an essential readjustment in the membranes of the evolved strain. Such rearrangement in the metabolism of lipids in ISO12 would be a coherent response towards both heat and hydrolysate-derived inhibitors during the long-term adaptation process, suggesting that at least some of the alterations in lipid composition could be essential for proper membrane development and function. The distinctions in lipid-related ORFs and lipid composition could also be connected with other biological functions of lipids related to stress response such as their role as signaling molecules during heat stress [107], their involvement during the initiation of translation of heat shock proteins [108, 109] and exocytosis, a process that also largely depends on lipid-related reactions [110] and is largely affected during cellular stress [111, 112].

### Physiological characterisation of ISO12

#### Growth on non-fermentable substrates and multicellularity

It has been established that in *S. cerevisiae*, the main targets of thermal death are located in the inner mitochondrial membrane [113] and that a loss of mitochondrial genes as well as the development of a mitochondria-defective (*petite)* phenotype are common effects of heat stress on yeasts [19, 113]. Growth evaluation of ISO12 and its parental strain ER was performed on media containing ethanol or glycerol as sole carbon sources since *petite* cells cannot grow in non-fermentable substrates [114, 115]. In ethanol, the biomass of ISO12 increased 6 times during the first 25 h (from OD620 = 0.6 to OD620 = 5.7 ± 0.2), while the biomass of ER increased around 9 times (from OD620 = 0.6 to OD620 = 9.3 ± 0.0). Growth was also observed on glycerol plates for the evolved strain (Fig. 2-d). The proportion of *rho*^*−*^ cells (i.e. *petite* mutants lacking or with defective mitochondrial DNA) could not be determined for any of the strains since there was no difference in colony size compared with the YPDG plates. Therefore, although ISO12 did not present a true *petite* phenotype, the reduced growth rate in ethanol and the slower growth under mild conditions previously reported [20] hinted towards the presence of alteration(s) within the respiratory chain [115]. The correlates to the changes in cytochrome c heme lyase (*CYC3*), that was one of the 11 genes whose evolution was predicted to have occurred under positive selective pressure (Additional file 6). As discussed above, changes to this gene have been related to respiratory deficiencies at the benefit of decreased ROS levels [76, 77] which are two relevant aspects with respect to the ISO12 phenotype.

Additionally, the evaluation of growth on plates revealed a phenotype that has been denoted as “colony morphology response” and that is used to describe colonies with complex and organized structures [116]. This phenotype was observed for ISO12 only (Fig. 2) and the specific pattern was dependent on the carbon source. For instance, a characteristic “Y” shaped agar invasion was observed on the bottom of the glucose plates (Fig. 2-b). On glycerol, agar invasion was not perceived, but the surface of the colony had a regular and complex pattern (Fig. 2-d). The formation of biofilm after long-term cultivations was also a phenotype observed only in ISO12 strain (data not shown). As mentioned previously, agar invasion and biofilm formation have been reported as a response to different environmental stresses [117–119]. The complexity of the morphological response can be explained by the multiple and interacting signalling pathways. For instance, the filamentous growth MAPK cascade and the Ras-cAMP-PKA pathway (for a review see [120]) have been shown to be the two key signalling pathways behind the colony morphology response [116]. However, other signalling networks such as the Target of Rapamycin (TOR) pathway [121] and Cell Wall Integrity (CWI) pathway take also part in the regulation [122]. Indeed, the transcription of at least *FLO11* (one of the ORFs subjected to positive selection according to the model) is regulated by the MAPK and cAMP signalling pathways [123] and changes in the expression levels of this gene strongly affect colony morphology [124]. Furthermore, the changes to *MTL1* and *GPR1* also emphasize that the MAPK and RAS-cAMP signalling in itself has been altered. Based on these results we hypothesize that the presence of genetic variants in different ORFs related with the aforementioned signalling pathways and possible interactions with other proteins (such as Flo11p) account for the morphological phenotypes displayed by ISO12. Moreover, the capacity of multicellular behaviour observed in ISO12 becomes an additional piece of evidence towards the relevance of multicellularity as an evolution strategy to improve competitiveness and tolerance to environmental stresses [125–127].

## Conclusions

From the results presented in the current study, the improved tolerance to combined stresses in ISO12 is shown to reside in multiple mechanisms requiring more than just a few mutations. The results indicate that the adaptive evolution that was used to generate ISO12 gave rise to non-synonymous variants significantly enriched in GO terms related to cell periphery-related proteins. It was also found that, besides the improved thermotolerance of ISO12 reported in the previous study, the evolved strain has also a higher capability than ER to withstand the toxic effects of high concentrations of spruce hydrolysate-derived inhibitors and that such capability is not related with furaldehyde-reduction capacity (Additional file 1). The analysis of the lipid composition of the cells indicates that significant rearrangements in the lipidome may also account for the superior phenotype of ISO12 under conditions of stress. Membranes would be feasible and convenient targets for adaptation since the lethal effect of both types of stressors (heat and hydrolysate) could be counteracted by a common mechanism. Thus, the ISO12 genotype and phenotype converges to suggest that the membranes and lipidome of *S. cerevisiae* may play an important role during evolutionary adaptation to high temperature in the presence of hydrolysate-derived inhibitors. The key mutations, however, are difficult to pinpoint due to the high degree of strain mutability imposed by the conditions of the evolution process. Based on a predictive model of genes under positive selection and on other genomic variations investigated, the genes *MTL1, FLO1/5/9/11, CYC3, GPR1, ADH7* and *ENA1-2* are strong candidates for being directly responsible for the phenotypic changes between the strains. Further sequencing of different clones from the adaptation experiment displaying the improved phenotype could be done in the future to narrow the number of key mutations related with the superior phenotype. However, from an applied point of view it would seem more reasonable to attempt to replace the mutations that are responsible for negative traits accumulated in ISO12: e.g. its slower growth rate and its reduced capacity for NADPH-dependent reduction.

## Availability of supporting data

The sequence reads from this project have been deposited at the NCBI Sequence Read Archive under the accessions SRR2002842 (ER) and SRR2002960 (ISO12). The assembly data set supporting the results of this article has been deposited at DDBJ/EMBL/GenBank under the accessions JWJK00000000 (ER) and LBNP00000000 (ISO12). The versions described in this paper are version JWJK01000000 and LBNP01000000.

## Additional files

Additional file 1: **Supplementary Information.** This file contains supplementary Materials & Methods and Results for the physiological characterisation of ISO12 and for the distribution of sequence variants in the two strains.

Additional file 2: **Synonymous and non-synonymous variants in Ethanol Red.** This file contains a list of the synonymous and non-synonymous SNPs and INDELs that were found to be unique to Ethanol Red compared to ISO12 (with a S288c background). The analysis was performed using the Variant Annotation Integrator the UCSC Genome Browser.

Additional file 3: **Synonymous and non-synonymous variants in ISO12.** This file contains a list of the synonymous and non-synonymous SNPs and INDELs that were found to be unique to ISO12 compared to Ethanol Red (with a S288c background). The analysis was performed using the Variant Annotation Integrator the UCSC Genome Browser.

Additional file 4: **ORFs that were matched to the significantly enriched Gene Ontology terms.** This file contains a list of all the ORFs that were matched to the significantly enriched Gene Ontology (GO) terms (analysed with the YeastMine function at *Saccharomyces* Genome Database).

Additional file 5: **ORFs related to vesicle mediated transport, lipid metabolic processes and mitochondria.** This file contains a list of all the ORFs that were matched to these three Ontology (GO) terms (analysed with the AmiGO 2 database).

Additional file 6: **K**_**a**_ **/K**_**s**_ **modelling of ISO12 versus ER.** This file contains the results of the modelling of the non-synonymous to synonymous substitution ratio (K_a_/K_s_).

Additional file 7: **Copy Number Variation assessment of the reference strain regions in ER and ISO12.** This file contains the results of the read mapping-based Copy Number Variation (CNV) analysis of the reference strain (S288c) regions of ER and ISO12.

Additional file 8: **Assessment of the non-reference strain regions in ER and ISO12, including Copy Number Variations.** This file contains the results of the CNV-analysis of the non-reference strain regions of ER and ISO12 based on the *de novo* assemblies of both strains, as well as a list of the unmapped contigs and their alignments.

Additional file 9: **Metabolome and differential analysis of the lipid species compared between ER and ISO12.** This file contains the dataset of the relative concentration of each lipid species (119 metabolites) on each biological sample of ER and ISO12, together with the differential analysis.

## Abbreviations

CNV: Copy number variation
CWI: Cell wall integrity
ER: Ethanol Red
GO: Gene ontology
HMF: 5-(hydroxymethyl) furfural
HPLC: High performance liquid chromatography
INDEL: Insertion or deletion
MAPK: Mitogen-activated protein kinase
MLR: Maximal lysis rate
NGS: Next generation sequencing
OD: Optical density
ORF: Open reading frame
SGD: *Saccharomyces* genome database
SNP: Single nucleotide polymorphism
