# Supplementary material for: Cell periphery-related proteins as major genomic targets behind the adaptive evolution of an industrial Saccharomyces cerevisiae strain to combined heat and hydrolysate stress
Source: BMC Genomics. 2015 Jul 9;16(1):514. doi: 10.1186/s12864-015-1737-4 (PMC4496855; doi:10.1186/s12864-015-1737-4)
Supplement: Supplementary file 1 — Supplementary Information. This file contains supplementary Materials & Methods and Results for the physiological characterisation of ISO12 and for the distribution of sequence variants in the two strains. [file 12864_2015_1737_MOESM1_ESM.pdf]

Supplementary Information

Supplementary Materials & methods

Characterisation of the genome-level effect of the ISO12-adaptation

Analysis of the distribution of the sequence variants

The distribution of the sequence variants in ISO12 across the linearized S288c reference genome [1] was visualised with MATLAB (R2013a) [2], using the *hist* function with a 1 kb histogram bin size.

Physiological characterisation

Evaluation of cell viability and metabolic activity in undiluted spruce hydrolysate

The non-detoxified liquid fraction of steam-pretreated spruce (SEKAB, Örnsköldsvik, Sweden) was used during the experiments. The inhibitors identified in the hydrolysate were acetate (4.06 g. L^{-1}), 5-(hydroxymethyl) furfural (HMF; 1.07 g. L^{-1}) and furfural (0.95 g. L^{-1}). After pH adjustment with KOH pellets, the hydrolysate was supplemented with 20 g. L^{-1} glucose and 6.7 g. L^{-1} YNB (both added as powder). Pre-cultures of ER and ISO12 were grown overnight in 5 mL YPD medium (20 g. L^{-1} glucose, 10 g. L^{-1} yeast extract, 20 g. L^{-1} peptone) in 50 mL conical tubes. The pre-cultures were used to inoculate conical tubes containing 10 mL of 100% (v/v) spruce hydrolysate at low initial OD620 (~0.3-0.4). The cells were cultivated at 30°C and OD620 was measured at nine different points in time up to 72 hours. Cell viability was determined using the plate count method [3]: serial dilutions of the cell culture samples were plated on solid YPD and incubated at 30°C and after 48h of incubation, colonies were counted. The number of viable colony forming units was obtained per mL. A death rate (k) defined as the slope of the decrease of viable cells over time was calculated.

Metabolic activity was monitored by changes in the cultivation medium. Concentrations of glucose, glycerol, acetate, ethanol, HMF and furfural were determined at 0h 8h, 24h and 48h by HPLC as described above. The experiment was performed in biological triplicates.

Lyticase assay

Susceptibility to cell wall lysis was carried out according to Ovalle and collegues [4] with slight modifications. The assay was performed using lyophilized powder of lyticase (β-1,3 glucanase) from *Arthrobacter luteus* (≥2,000 U.mg protein^{-1}, Sigma-Aldrich, St. Louis, MO, USA). The lyticase was dissolved in cold, sterile TE-buffer (0.05 M Tris-HCl, 0.15 M NaCl, 5mM EDTA, adjusted to pH 7.5 with 3 M NaOH) to a concentration of 0.8 mg enzyme.mL^{-1}, corresponding to an activity of 1,600 U.mL^{-1}. YPD-grown cells at 30°C or 40°C were harvested during exponential phase and washed twice with deionized water and resuspended in TE buffer (25°C, pH= 7.5). The assay was performed with 3 mL of resuspended cells (~2x107 cells.mL^{-1}) during which the change in optical density at 660 nm (OD660) was followed every 10 minutes for up to 1 hour after addition of 100 µL of the enzyme suspension. The maximal lysis rate (MLR) [4] was defined, in this work, as the slope of the decrease in OD660 over time of incubation under the described conditions. The experiment was performed at least in three biological replicates.

Supplementary Results and discussion

Characterisation of the genome-level effect of the ISO12-adaptation

Analysis of the distribution of the sequence variants

As mentioned in the main paper, the variants in ER and ISO12 were found to be distributed throughout the (linearized) S288c reference genome, however with varying peak intensities (the variant distribution in ISO12 is illustrated in Figure S1). This pattern was still prevalent after the shared variants were subtracted and many of the peaks with highest density were found in intergenic regions (ISO12: Figure S1B and C). We could also observe a trend that the variant counts were peaking towards the chromosomal ends (ISO12: Figure S1); however, this could be explained by the fact that telomeric regions are difficult to sequence, and thus also correctly assess for variants, due to the high content of tandem repeats [5].

Physiological characterisation of ISO12

Spruce hydrolysate and cell viability

In the previous study, it was shown that the tolerance towards hydrolysate-derived inhibitors – measured by the duration of the lag phase and the growth rate in the presence of 50% (v/v) spruce hydrolysate – was not different between the strains, and that the capacity of ISO12 for NADPH-dependent reduction of HMF was actually diminished [6]. In order to evaluate whether ISO12 had developed an alternative mechanism for tolerance towards hydrolysate-derived inhibitors, low cell concentrations (OD620~0.3-0.4) were exposed to 100% (v/v) spruce hydrolysate. Under these conditions, none of the strains showed growth (data not shown). However, the loss in viability of ER cultures was faster than that of ISO12 cultures (Figure S2). The death rate obtained for ER during the first eight hours ($k= 0.82 \pm 0.27\ h^{-1}$) was approximately four times higher than the death rate of ISO12 ($k= 0.21 \pm 0.11\ h^{-1}$) ($n=3$; $p<0.05$). No reduction of HMF was observed in any of the cultures after 48h while furfural was only slightly reduced by ISO12 cultures (Table S1). This is consistent with previous evidence showing that when both furaldehydes were present, HMF reduction occurred only after furfural has been reduced and at a slower rate [7, 8].

The absence of growth, the only slight decrease in furfural concentration, together with the rapid loss in viability indicated that the cells could not detoxify HMF and only slightly reduce furfural during the 72 h of the experiment. But the lower death rate observed for ISO12 cultures suggested that the cells could cope for longer time with the toxic effects of the inhibitors. This phenotype is coherent with the sequencing and lipidome results, and reinforces the hypothesis that the higher tolerance to inhibitors in ISO12 relies on changes in the permeability of the cells. Reduced cell permeability as a result of changes in the composition of the plasma membrane has in fact been previously reported as a trait in *S. cerevisiae* strains with improved tolerance towards furfural, phenol, acetic acid and ethanol [9, 10]. An increased capacity of the ISO12 cells to expel the inhibitors, by for example alterations in the activity of efflux transporters, cannot be discarded though.

Cell wall susceptibility to lyticase

To investigate whether the improved thermotolerance of ISO12 could rely on differences in the cell wall composition, the sensitivity of both strains to lyticase (β-1,3-glucanase) was evaluated as lyticase acts on β 1,3-glucan, the major component of the cell wall in *S. cerevisiae* , together with chitin and mannoproteins [11]. The maximal lysis rates (MLR) of ER and ISO12 cultures grown at the same temperature (30°C or 40°C) were not significantly different between the strains ($p>0.1$) (Table S2 and Figure S3). In contrast, the MLRs of both strains were affected by the cultivation temperature, with cultures grown at 30°C having MLRs approximately 8 times higher than rates observed for cultures grown at 40°C (Table S2).

The effect of the cultivation temperature on the composition of the main cell wall constituents was reported by Aguilar and co-workers [12]. In the study, decreased mannan content and increased concentration of chitin and β-glucans were measured for cells grown at 37°C compared with cells grown at 30°C. The maximal lysis rate was higher for the cultures grown at the lower temperature, which is consistent with the results observed for ER and ISO12. Altogether, the results indicate that high temperature (in this case 40°C) indeed stimulates the reinforcement of the cell wall through changes in β 1,3-glucan in ER and ISO12, but that the evolution mechanisms behind the improved thermotolerance in ISO12 was not a result of structural changes in β 1,3-glucan. To elucidate whether possible changes in other cell wall components may account for the differences in performance of ISO12 and ER, further analysis would be required.

Supplementary Figures

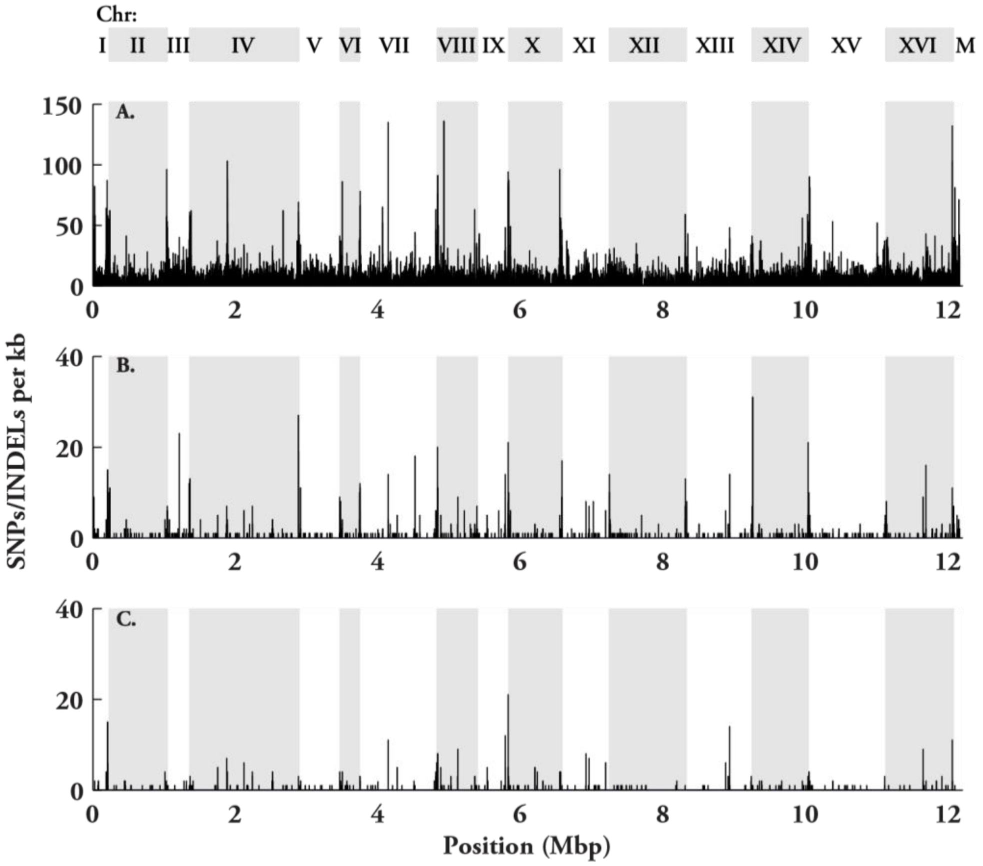

Figure S1. Distribution of the ISO12 variants (SNPs and INDELs) across the linearized *S. cerevisiae* genome using a 1 kb histogram bin size. The alternating white-grey rectangles represent the positions of the different chromosomes (Chr). A: Distribution of all variants detected against S288c; B: Unique variants remaining in ISO12 after subtraction of all variants common to both Ethanol Red and ISO12; C: Unique variants in ISO12 filtered to only display variants that occur in coding regions.

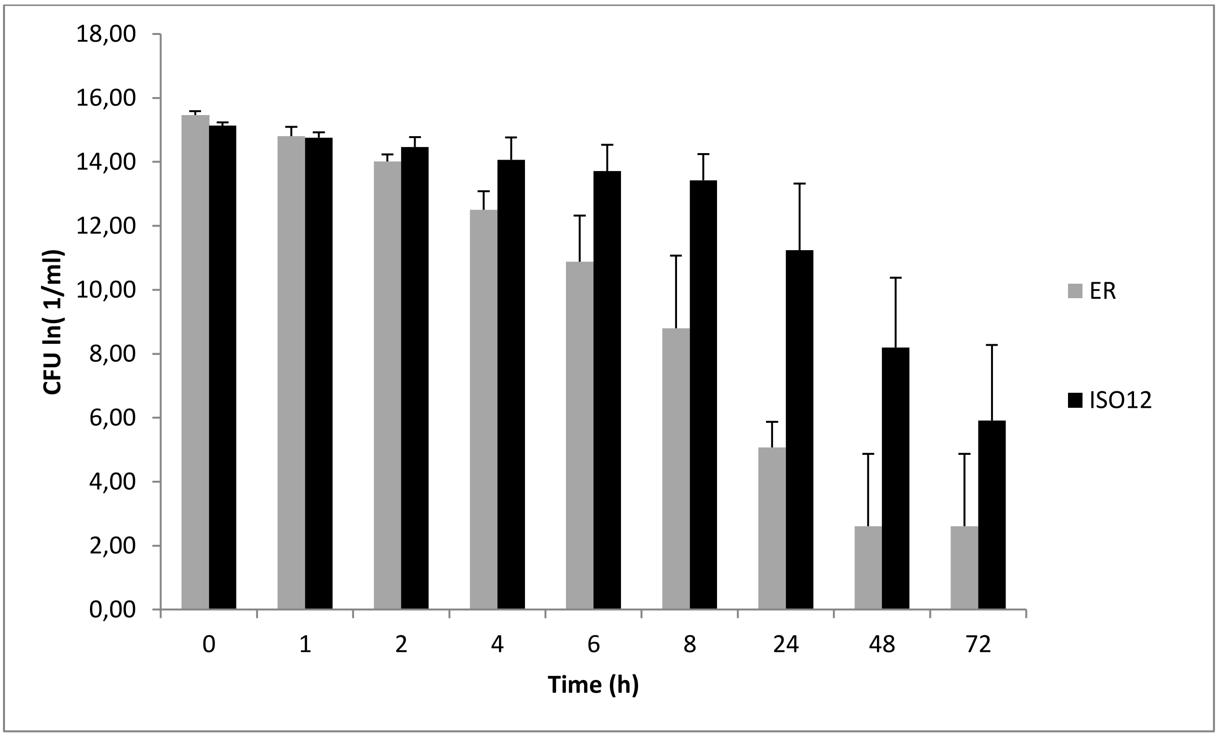

Figure S2. Cell viability of ER (grey) and ISO12 (black) during growth on 100% (v/v) spruce hydrolysate, 30 °C. The experiments were performed in biological triplicates. The bars indicate the deviation from the mean.

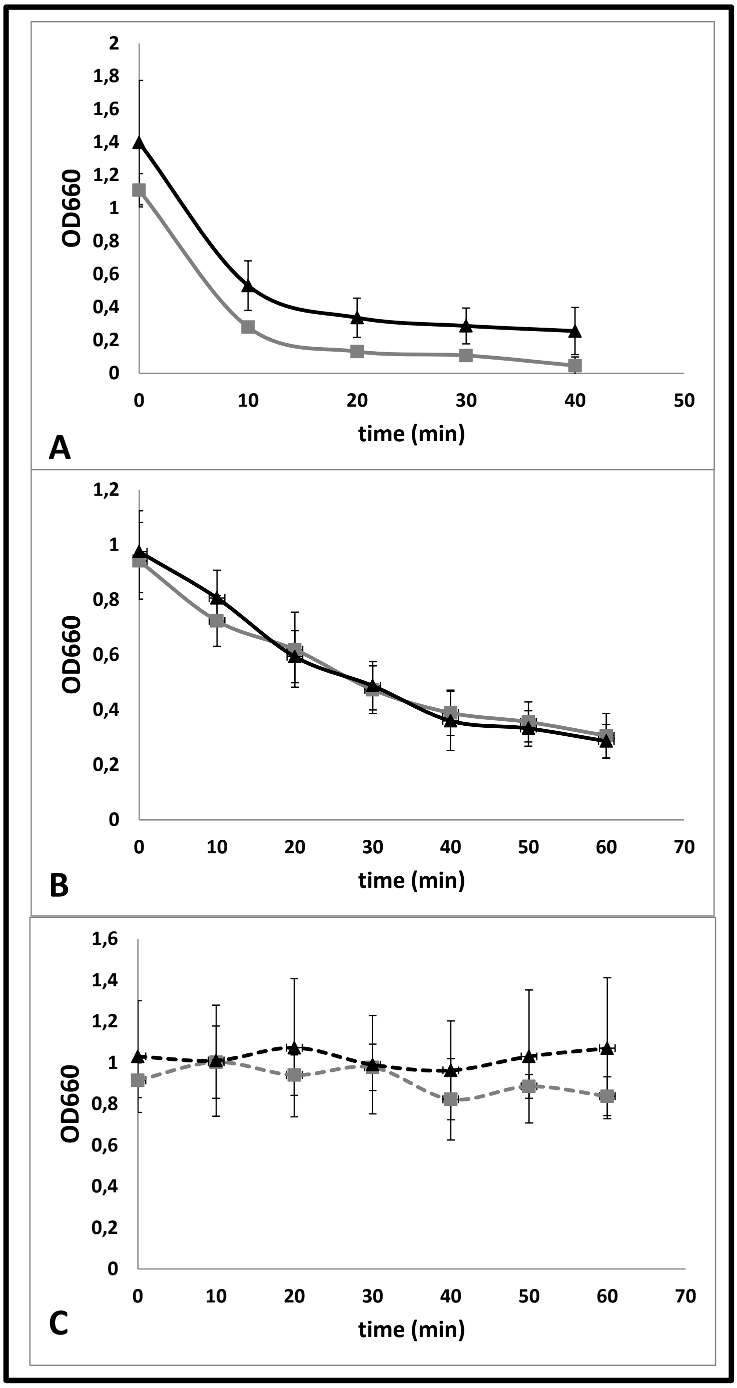

Figure S3. Lysis profile of ER (gray) and ISO12 (black) of cultures grown in YPD at 30°C (A) or 40°C (B). Profile in C shows control conditions (no lyticase). The figures show the average profile of three biological replicates. The bars indicate deviation from the mean.

Supplementary Tables

Table S1. HPLC analysis of the hydrolysate-based media used for cell cultivations with the average metabolite concentrations during the course of the hydrolysate viability experiment. The experiments were performed in biological triplicates. The hydrolysate without added glucose and YNB was pH adjusted to 5.5.

**Ethanol Red**	**Glucose**	**Glycerol**	**Acetate**	**Ethanol**	**HMF**	**Furfural**
0 h	29,17 ± 0,01	0,48 ±0,01	3,79 ±0,12	0,28 ±0,09	0,97 ±0,03	0,87 ±0,03
8 h	28,16 ±1,10	0,47 ±0,01	3,73 ±0,05	0,35 ±0,09	0,93 ±0,02	0,79 ±0,03
24 h	30,49 ±0,56	0,52 ±0,03	4,20 ±0,18	0,42 ±0,12	1,03 ±0,05	0,85 ±0,04
48 h	30,03 ±0,01	0,50 ±0,02	4,09 ±0,15	0,33 ±0,13	0,99 ±0,10	0,77 ±0,10
**ISO12**	**Glucose**	**Glycerol**	**Acetate**	**Ethanol**	**HMF**	**Furfural**
0 h	29,48 ±0,84	0,49 ±0,03	3,98 ±0,12	0,16 ±0,11	1,03 ±0,04	0,91 ±0,03
8 h	28,32 ±0,56	0,47 ±0,01	3,75 ±0,03	0,32 ±0,10	0,95 ±0,01	0,75 ±0,01
24 h	28,47 ±1,25	0,44 ±0,12	4,04 ±0,32	0,51 ±0,14	1,00 ±0,09	0,80 ±0,16
48 h	32,76 ±4,82	0,54 ±0,05	4,33 ±0,48	0,61 ±0,10	1,05 ±0,12	0,68 ±0,13
**Hydrolysate w.o. glucose & YNB**	**Glucose**	**Glycerol**	**Acetate**	**Ethanol**	**HMF**	**Furfural**
	10,86 ±0,10	0,49 ±0,01	4,06 ±0,02	0,00 ±0,00	1,07 ±0,01	0,95 ±0,02

Table S2. Effect of the cultivation temperature on the sensitivity of cells to lyticase. Values show the average of 3 biological replicates ±standard deviation.

Strain	Maximal rate lysis (min^{-1})	
	Cells grown at 30°C[a]	Cells grown at 40°C[b]
ER	0.083 ± 0.005	0.010 ± 0.001
ISO12	0.089 ± 0.023	0.011 ± 0.003

a: Measured during the first 10 minutes; b: Measured during the first 60 minutes

References

1. Engel SR, Dietrich FS, Fisk DG, Binkley G, Balakrishnan R, Costanzo MC, Dwight SS, Hitz BC, Karra K, Nash RS, Weng S, Wong ED, Lloyd P, Skrzypek MS, Miyasato SR, Simison M, Cherry JM: **The Reference Genome Sequence of *Saccharomyces cerevisiae*: Then and Now**. *G3-Genes Genom Genet* 2014, **4**(3):389-398.
2. MathWorks: **MATLAB Release 2013a.** *The MathWorks Inc, Natick, Massachusetts, United States* 2013.
3. Adams MR, Moss MO: **Methods for the Microbiological Examination of Foods**. In: *Food Microbiology.* 3rd edn. Cambridge: The Royal Society of Chemistry; 2008: 370-395.
4. Ovalle R, Lim ST, Holder B, Jue CK, Moore CW, Lipke PN: **A spheroplast rate assay for determination of cell wall integrity in yeast**. *Yeast* 1998, **14**(13):1159-1166.
5. Cohn M, Liti G, Barton DB: **Telomeres in fungi**. In: *Comparative Genomics.* Springer; 2006: 101-130.
6. Wallace-Salinas V, Gorwa-Grauslund MF: **Adaptive evolution of an industrial strain of *Saccharomyces cerevisiae* for combined tolerance to inhibitors and temperature**. *Biotechnol Biofuels* 2013, **6**(1):151.
7. Larsson S, Palmqvist E, Hahn-Hägerdal B, Tengborg C, Stenberg K, Zacchi G, Nilvebrant NO: **The generation of fermentation inhibitors during dilute acid hydrolysis of softwood**. *Enzyme Microb Tech* 1999, **24**(3-4):151-159.
8. Taherzadeh MJ, Gustafsson L, Niklasson C, Lidén G: **Physiological effects of 5-hydroxymethylfurfural on *Saccharomyces cerevisiae***. *Appl Microbiol Biot* 2000, **53**(6):701-708.
9. Xia JM, Yuan YJ: **Comparative Lipidomics of Four Strains of *Saccharomyces cerevisiae* Reveals Different Responses to Furfural, Phenol, and Acetic Acid**. *J Agr Food Chem* 2009, **57**(1):99-108.
10. Kim HS, Kim NR, Choi W: **Total fatty acid content of the plasma membrane of *Saccharomyces cerevisiae* is more responsible for ethanol tolerance than the degree of unsaturation**. *Biotechnol Lett* 2011, **33**(3):509-515.
11. Orlean P: **Architecture and Biosynthesis of the *Saccharomyces cerevisiae* Cell Wall**. *Genetics* 2012, **192**(3):775-818.
12. Aguilar-Uscanga B, Francois JM: **A study of the yeast cell wall composition and structure in response to growth conditions and mode of cultivation**. *Lett Appl Microbiol* 2003, **37**(3):268-274.
